# A Comprehensive Assessment of the Precision and Agreement of Anterior Corneal Power Measurements Obtained Using 8 Different Devices

**DOI:** 10.1371/journal.pone.0045607

**Published:** 2012-09-25

**Authors:** Qinmei Wang, Giacomo Savini, Kenneth J. Hoffer, Zhen Xu, Yifan Feng, Daizong Wen, Yanjun Hua, Feng Yang, Chao Pan, Jinhai Huang

**Affiliations:** 1 Department of Ophthalmology, School of Optometry and Ophthalmology and Eye Hospital, Wenzhou Medical College, Wenzhou, Zhejiang, China; 2 Key Laboratory of Vision Science, Ministry of Health P.R. China, Wenzhou, Zhejiang, China; 3 Department of Ophthalmology, G.B. Bietti Eye Foundation-IRCCS, Rome, Italy; 4 Department of Ophthalmology, Jules Stein Eye Institute, University of California Los Angeles, Los Angeles, California, United States of America; 5 Department of Ophthalmology, Eye Center, The 180th Hospital of People's Liberation Army, Quanzhou, Fujian, China; Medical University Graz, Austria

## Abstract

**Purpose:**

To comprehensively assess the precision and agreement of anterior corneal power measurements using 8 different devices.

**Methods:**

Thirty-five eyes from 35 healthy subjects were included in the prospective study. In the first session, a single examiner performed on each subject randomly measurements with the RC-5000 (Tomey Corp., Japan), KR-8000 (Topcon, Japan), IOLMaster (Carl Zeiss Meditec, Germany), E300 (Medmont International, Australia), Allegro Topolyzer (Wavelight AG, Germany), Vista (EyeSys, TX), Pentacam (Oculus, Germany) and Sirius (CSO, Italy). Measurements were repeated in the second session (1 to 2 weeks later). Repeatability and reproducibility of corneal power measurements were assessed based on the intrasession and intersession within-subject standard deviation (Sw), repeatability (2.77Sw), coefficient of variation (COV), and intraclass correlation coefficient (ICC). Agreement was evaluated by 95% limits of agreement (LoA).

**Results:**

All devices demonstrated high repeatability and reproducibility of the keratometric values (2.77Sw<0.36D, COV<0.3%, ICC>0.98). Repeated-measures analysis of variance with Bonferroni post test showed statistically significant differences (*P*<0.01) among mean keratometric values of most instruments; the largest differences were observed between the EyeSys Vista and Medmont E300. Good agreement (i.e., 95%LoA within ±0.5D) was found between most instruments for flat, steep and mean keratometry, except for EyeSys and Medmont. Repeatability and reproducibility of vectors J_0_ and J_45_ was good, as the ICCs were higher than 0.9, except J_45_ of Medmont and Pentacam. For the 95% LoAs of J_0_ and J_45_, they were all ≤ ±0.31 among any two paired devices.

**Conclusions:**

The 8 devices showed excellent repeatability and reproducibility. The results obtained using the RC-5000, KR-8000, IOLMaster, Allegro Topolyzer, Pentacam and Sirius were comparable, suggesting that they could be used interchangeably in most clinical settings. Caution is warranted with the measurements of the EyeSys Vista and Medmont E300, which should not be used interchangeably with other devices due to lower agreement.

**Trial Registration:**

ClinicalTrials.gov NCT01587287

## Introduction

The ability to determine corneal curvature with a high degree of accuracy and reliability is important in both clinical and research conditions. Corneal curvature measurement provides crucial information for calculating intraocular lens (IOL) power, [1] screening and managing corneal refractive surgeries, [2], [3] designing, monitoring and assessing the fit of orthokeratology and customized contact lenses. [4] Although a number of studies have been performed to investigate the reliability and accuracy of corneal power measurements obtained using different technologies, such as manual or automated keratometry, computerized videokeratography, raster-stereogrammetry, slit-scanning tomography, rotating Scheimpflug tomography and optical coherence tomography,[5]–[21] to the best of the our knowledge, no study has assessed the intrasession and intersession precision as well as the interchangeability of corneal power measurements acquired by 8 different devices (three autorefractor keratometers (Tomey RC-5000 (Tomey Inc., Nagoya, Japan), Topcon KR-8000 (Topcon Corp., Tokyo, Japan), IOLMaster (Carl Zeiss, Jena, Germany)), three Placido disk–based corneal topographers (Medmont E300 (Medmont Pty. Ltd., Victoria, Australia), Allegro Topolyzer (WaveLight Technologie AG [Alcon Laboratories], Erlangen, Germany), EyeSys Vista (EyeSys INC., Texas, USA)), a single-rotating Scheimpflug imaging device (Pentacam, Oculus, Wetzlar, Germany), and a new single-rotating-Scheimpflug-single-Placido hybrid analyzer (Sirius, Costruzione Strumenti Oftalmici, Florence, Italy) on the same eye under the same clinical setting.

The present study sought to prospectively determine the intrasession repeatability and intersession reproducibility of anterior corneal curvature measurements by using the 8 commercially available instruments mentioned above and evaluate differences in mean corneal power measurements for each of the different instruments in order to check the agreement between devices.

## Subjects and Methods

The protocol for this trial and supporting CONSORT checklist are available as supporting information; see Checklist S1 and Protocol S1.

### Subjects

Thirty-five young adult subjects (10 males and 25 female) with a mean age of 24.60±1.64 years (range 21 to 28 years), the mean manifest spherical equivalent refraction was −4.15±2.06 diopters (range, −1.0 to −9.0 diopters), were recruited for this prospective study. All procedures followed the Declaration of Helsinki, and the protocol was reviewed and approved by the Research Review Board at Wenzhou Medical College. The written informed consent was received from all subjects before inclusion in the study. All subjects had good best corrected distance visual acuity (BCVA) equal to or better than 20/25 to allow for adequate fixation. The exclusion criteria were 1) intellectual disability or somatic dysfunction, 2) previous ocular surgery, 3) history of ocular pathology, 4) contact lens wearers, and 5) dry eye (significant subjective dry eye symptoms, Schirmer I test results of less than 5.0 mm, tear film break-up time shorter than 5 seconds and corneal fluorescein staining positive. All of these conditions can result in abnormal measurements. Each subject underwent a full ophthalmic examination including vision, auto- and subjective refraction, slit-lamp examination, non-contact tonometry, corneal power measurements with the 8 devices presented above and fundus examination.

### Instruments

The Tomey RC-5000 (software version 1.2.6 ) and the Topcon KR-8000 (software version Release 2E) Autorefractors are designed based on the optical principle represented by the relationship between the size of an object and the size of the image of that object reflected from a surface. Assuming the cornea is a convex mirror, the automated keratometer instantly records the size and computes the radius of curvature while focusing the reflected corneal image (infrared illuminated mires) onto an electronic photosensitive device (infrared detectors). Both devices acquire radius of curvature measurements in the flat and steep meridians on a 3.0-mm diameter field of the central cornea.

The IOLMaster (software version 5.4) also works according to the optical principle represented by the reflection by the anterior surface of a luminous pattern of mires in the center of the cornea. It uses data from a hexagonal array of 6 points reflected off the surface of the cornea, which depends on the corneal curvature. To calculate corneal curvature, the IOLMaster reflects six points of light, arranged in a 2.5 mm-diameter hexagonal pattern, from the air/tear film interface.

The Medmont E-300 (software version 5.1.0) is a Placido disk-based videokeratoscope that utilizes an arc-step reconstruction algorithm and incorporates a range finder. [5], [22] It determines the distance from the corneal apex to the instrument’s camera and automatically captures images. It has 32 Placido rings and measures 9 600 data points per scan. Each image captured is awarded a score out of 100 based on centering, focus and movement. The images were selected and saved when good focus and alignment were attained. A score higher than 75 was considered good. The device acquires radius of curvature measurements in the flat and steep meridians on a 3.0-mm diameter field of the central cornea.

The Allegro Topolyzer (software version 1.59) and EyeSys Vista (software version 3.11) are also Placido disk-based videokeratoscopes. The former contains 22 rings and measures and generates high-resolution data of the corneal surface with 22 000 data points; the latter allows for freedom and portable corneal topography and is incorporated into the iTrace system with integrated software. The device contains 26 Placido rings and measures 9 360 points. Both devices present keratometric data in three corneal zones: a central zone with a 3-mm diameter, a paracentral zone with a 5-mm diameter, and a peripheral zone with a 7-mm diameter. In this study, the 3-mm zone readings were chosen for improved correlation with the central optical zone and the areas of measurement covered by other instruments.

The most recent version of the Pentacam-HR rotating Scheimpflug camera system (software version 1.17r89) was used in this study. It captures 138,000 true elevation points using a high-resolution, 1.45 mega-pixel camera. The automatic release mode was used to reduce the number of operator-dependent variables. In less than 2 seconds, the rotating camera obtains 25 slit images of the anterior segment. Only scans with an “Examination Quality Specification” of “OK” were chosen for analysis.

The Sirius is a new device that combines the use of single-Scheimpflug cameras and a Placido disk to measure and image the anterior eye segment, including the cornea, anterior chamber, iris, pupil, and lens. It can acquire 25 Scheimpflug frames and one keratoscopy reading in less than 1 second. It is capable of measuring anterior and posterior tangential (instantaneous) curvature, sagittal (axial) curvature altimetry and refractive power, equivalent refractive power, corneal thickness, and visual quality (spot diagram, point-spread function and optical transfer function). Anterior corneal measurements are performed by the Sirius using a proprietary method of merging the Placido and Scheimpflug data. The corneal power was calculated by averaging the axial curvature from the 4th to the 8th Placido ring. [6] Only scans with an “image acquisition quality” of “Scheimpflug images Coverage ≥90%, Centration ≥90%, Keratoscopy Coverage ≥80%” were chosen for analysis by the available software version 1.0. Both Scheimpflug camera systems acquire radius of curvature measurements in the flat and steep meridians on a 3.0-mm diameter field of the central cornea.

All instruments convert the curvature measurements obtained from the anterior corneal surface into a total corneal dioptric value using the thin lens formula (n_1_−n_0_)/r, where n_0_ = refractive index of air ( = 1.0000) and an n_1_ = refractive index of the cornea ( = 1.3375) and r = radius in mm.

### Procedures

The present study’s definitions of reproducibility, repeatability and agreement were based on those adopted by the British Standards Institute and the International Organization for Standardization. [23]–[25] The testing sequence of the measurements with these devices was randomly chosen to avoid methodological bias. MedCalc Statistical Software version 10.0.1.0 (MedCalc Software, Inc., Mariakerke, Belgium), predetermined generate random sample program. The measurements were collected at least 3 hours after subjects woke from sleep. The subjects were asked to avoid substantial reading prior to the measurements. [26] All measurements were conducted between 10 am and 5 pm to minimize variations in the results. Only the right eye of each subject was selected, and cycloplegic drugs were not used. During the first session, three sets of measurements with all the devices were performed by a single experienced examiner (X.Z.) for all subjects according to the manufacturers’ instructions. The examiner and subject were masked to the results of the previous measurements obtained from each device. Subjects were instructed to blink completely just before each measurement. The subjects were asked to sit back after each repeat measurement, and the device was realigned before each measurement. The time between repeated scans by the observer was the minimum possible, and the measurements among different instruments were continuous, without significant time intervals. Measurements were repeated in the second session scheduled 1 to 2 weeks later, at almost the same time as the first session, by the same examiner using the same protocol (i.e., 3 measurements with each device). Intersession reproducibility was determined as well. The mean of the 3 measurements of the first session was calculated for each non-contact keratometry device to assess the agreement among the 8 methods.

### Sample Size Estimation

Sample size calculation was performed a priori using PS Power and Sample Size Calculation Software (version 3.014, Vanderbilt University, Tennessee, USA). Based on the result of a recent study of corneal power measurements obtained by different devices, the pooled SD of the differences in keratometry between devices was approximately 0.12 diopters (D). [7] Using a two-sided level of level of significance (α) = 5% and a power (1−β) = 99%, a sample size of 29 eyes as a requirement to detect a difference of 0.10 D between instruments.

### Statistical Analysis

Statistical analysis was performed using SPSS software for Windows version 13 (SPSS Inc., Chicago, IL, U.S.) and Microsoft Office Excel. A P value of less than 0.05 was considered to be statistically significant. The distributions of the datasets were checked for normality using Kolmogorov–Smirnov tests. The results indicated that the data were normally distributed (*P*>.05). For each measurement, the flat (Kf) and steep (Ks) corneal power values, the average power of Kf and Ks (Kave), and the axes of Kf and Ks were noted. The corneal astigmatism was converted into a vector representation, J_0_ (cylinder at 0-degree meridian) and J_45_ (cylinder at 45-degree meridian), which were calculated according to the following formulas: [27].

J0 = − [cylinder/2] cos[2 × axis];

J45 ( = − [cylinder/2] sin[2 × axis]).

These values were calculated for 3 separate measurements in each session and then averaged to determine the reproducibility and the comparability elevation.

### Intrasession Repeatability and Intersession Reproducibility

To determine the intrasession repeatability of each device, within-subject standard deviation (S_w_), test-retest repeatability (TRT), the within-subject coefficient of variation (COV), and intraclass correlation coefficients (ICC) were calculated for the three repeated measurements obtained during the first and second sessions. [28] TRT was defined as 2.77 S_w_, which means an interval within which 95% of the differences between measurements are expected to lie. The COV was calculated as the ratio of the Sw to the overall mean. A lower COV is associated with higher repeatability.

The advantage of COV values is that they can be compared between data sets with different units or widely different means. The disadvantage is that when the mean value is near zero, the COV is sensitive to small changes in the mean, limiting its usefulness. Therefore we did not calculate the COV for both vector J_0_ and J_45_, whose mean values are close to zero. [8], [9] The ICCs (ranging from 0 to 1) measure the consistency for data sets of repeated measurements. The closer the ICC is to 1, the better the measurement consistency. To assess intersession reproducibility, the mean of the three readings from each session was firstly calculated for each device, and then intersession S_w_, 2.77 S_w_, COV and ICCs were also calculated.

### Comparison Among Devices

Repeated-measures analysis of variance (ANOVA) with Bonferroni correction was used to identify pairs that were significantly different. Bland-Altman analysis was performed to evaluate the agreement between devices. This involved the use of the 95% limits of agreement (LoA) as the mean difference ±1.96 SD. A narrower 95% LoA indicates superior agreement between techniques.

## Results

### Intrasession Repeatability

For the repeatability during the first and second sessions, the 2.77 S_ws_ of repeated Kf and Ks measurements were lower than 0.36 D. With all devices, the COV was lower than 0.3% and the ICC higher than 0.98 (Tables 1 and 2). The COV of Kave was lower than 0.26%, and the ICC higher than 0.99 in both sessions (Table 3). For vectors J_0_ and J_45_, during the first session, the 2.77S_ws_ values were lower than 0.36, and the ICCs were higher than 0.94, except for J_45_ on the Medmont (0.747) and J_45_ on the Pentacam (0.85). The second session displayed a similar tendency; the 2.77S_w_ were lower than 0.27, and the ICCs were higher than 0.92, except for J_45_ on the Medmont (0.844) and J_45_ on the Pentacam (0.86) (Tables S1 and S2).

**Table 1:** Intrasession Repeatability of 8 Different Devices in Measuring Flat Keratometry (N = 35).

Device	Session	Mean (D) ± SD	Sw (D)	2.77 Sw (D)	COV (%)	ICC
Tomey RC	1st	42.85±1.26	0.04	0.10	0.08	0.999
	2nd	42.85±1.27	0.03	0.09	0.08	0.999
Topcon KR	1st	42.90±1.30	0.05	0.15	0.13	0.998
	2nd	42.91±1.27	0.06	0.17	0.14	0.998
IOLMaster	1st	42.97±1.27	0.03	0.09	0.07	0.999
	2nd	42.99±1.27	0.04	0.10	0.08	0.999
EyeSys Vista	1st	42.65±1.21	0.13	0.36	0.30	0.989
	2nd	42.64±1.19	0.10	0.29	0.24	0.992
Medmont	1st	43.05±1.29	0.07	0.20	0.17	0.997
	2nd	43.06±1.27	0.07	0.20	0.17	0.997
Topolyzer	1st	42.90±1.28	0.10	0.27	0.23	0.994
	2nd	42.91±1.25	0.10	0.27	0.23	0.994
Pentacam	1st	42.90±1.26	0.08	0.21	0.18	0.996
	2nd	42.90±1.26	0.07	0.20	0.17	0.997
Sirius	1st	42.97±1.26	0.10	0.27	0.23	0.994
	2nd	43.01±1.28	0.08	0.22	0.18	0.996

D = diopter, SD = standard deviation, Sw = within-subject standard deviation, COV = within-subject coefficient of variation, ICC = intraclass correlation coefficient.

**Table 2:** Intrasession Repeatability of 8 Different Devices in Measuring Steep Keratometry (N = 35).

Device	Session	Mean (D) ± SD	Sw (D)	2.77 Sw (D)	COV (%)	ICC
Tomey RC	1st	43.87±1.44	0.05	0.14	0.12	0.999
	2nd	43.86±1.46	0.04	0.12	0.10	0.999
Topcon KR	1st	43.74±1.43	0.08	0.21	0.17	0.997
	2nd	43.76±1.44	0.08	0.23	0.19	0.997
IOLMaster	1st	43.97±1.44	0.04	0.11	0.09	0.999
	2nd	43.96±1.43	0.05	0.15	0.12	0.999
EyeSysVista	1st	43.53±1.38	0.12	0.34	0.28	0.992
	2nd	43.54±1.39	0.13	0.35	0.29	0.992
Medmont	1st	44.08±1.44	0.07	0.20	0.16	0.998
	2nd	44.15±1.46	0.08	0.23	0.18	0.997
Topolyzer	1st	43.84±1.46	0.13	0.35	0.29	0.993
	2nd	43.84±1.45	0.12	0.32	0.27	0.994
Pentacam	1st	43.85±1.43	0.09	0.24	0.19	0.996
	2nd	43.86±1.45	0.08	0.23	0.19	0.997
Sirius	1st	43.90±1.46	0.10	0.29	0.24	0.995
	2nd	43.96±1.46	0.10	0.27	0.22	0.996

D = diopter, SD = standard deviation, Sw = within-subject standard deviation, COV = within-subject coefficient of variation, ICC = intraclass correlation coefficient.

**Table 3:** Intrasession Repeatability of 8 Different Devices in Measuring Mean Keratometry (N = 35).

Device	Session	Mean (D) ± SD	Sw (D)	2.77 Sw (D)	COV (%)	ICC
Tomey RC	1st	43.36±1.31	0.03	0.08	0.06	1.000
	2nd	43.36±1.33	0.03	0.07	0.06	1.000
Topcon KR	1st	43.32±1.34	0.06	0.16	0.13	0.998
	2nd	43.33±1.33	0.06	0.17	0.14	0.998
IOLMaster	1st	43.47±1.32	0.02	0.06	0.05	1.000
	2nd	43.48±1.32	0.03	0.07	0.06	1.000
EyeSysVista	1st	43.09±1.26	0.11	0.32	0.26	0.992
	2nd	43.09±1.26	0.10	0.29	0.24	0.993
Medmont	1st	43.57±1.33	0.07	0.18	0.15	0.999
	2nd	43.60±1.33	0.06	0.17	0.14	0.998
Topolyzer	1st	43.37±1.34	0.11	0.29	0.24	0.994
	2nd	43.37±1.32	0.10	0.28	0.23	0.994
Pentacam	1st	43.38±1.31	0.07	0.18	0.15	0.998
	2nd	43.38±1.32	0.06	0.17	0.14	0.998
Sirius	1st	43.44±1.33	0.09	0.25	0.21	0.996
	2nd	43.48±1.34	0.08	0.21	0.18	0.997

D = diopter, SD = standard deviation, Sw = within-subject standard deviation, COV = within-subject coefficient of variation, ICC = intraclass correlation coefficient.

### Intersession Reproducibility

Tables S3–7 show that the reproducibility of corneal power measurements was excellent for all devices. The differences between both sessions were lower than 0.06 D for each device comparison. The intersession reproducibility parameters demonstrated a trend similar to that of the intrasession repeatability assessments. The 2.77 S_w_ of repeated Kf and Ks measurements were lower than 0.35 D; the COV was lower than 0.28%, and the ICCs higher than 0.99 in all devices (Tables S3 and S4). The ICC was ≥0.99 also for Kave (Table S5). Though the ICC_s_ for power vectors J_0_ and J_45_ were lower than Kf and Ks, they were still higher than 0.9 except J_45_ of IOLMaster (0.898) and J_45_ of Medmont (Tables S6 and S7).

### Comparison between Devices

Tables 4, 5, 6 and Tables S8–9 list the mean difference, SD and 95% LoA for any paired comparison of the eight devices. The highest mean difference in Kf, Ks, Kave, J_0_ and J_45_ was 0.4 D, 0.55 D, 0.48 D, 0.11 and 0.11, respectively.

**Table 4:** Comparison of the Flat Keratometry between 8 Different Devices.

Devices	Mean Difference(D) ± SD	*P* Value	95% LoA (D)
Tomey-Topcon	−0.05±0.09	<.01	−0.226 to 0.134
Tomey-IOLMaster	−0.12±0.08	<.01	−0.278 to 0.044
Tomey-EyeSys	0.20±0.11	<.01	−0.010 to 0.41
Tomey-Medmont	−0.20±0.15	<.01	−0.480 to 0.090
Tomey-Topolyzer	−0.05±0.09	<.01	−0.231 to 0.133
Tomey-Pentacam	−0.05±0.09	<.01	−0.236 to 0.132
Tomey-Sirius	−0.12±0.11	<.01	−0.330 to 0.09
Topcon-IOLMaster	−0.07±0.09	<.01	−0.246 to 0.103
Topcon-EyeSys	0.24±0.14	<.01	−0.02 to 0.51
Topcon-Medmont	−0.15±0.14	<.01	−0.42 to 0.12
Topcon-Topolyzer	0.00±0.10	.851	−0.196 to 0.19
Topcon-Pentacam	−0.01±0.11	.735	−0.22 to0.21
Topcon-Sirius	−0.08±0.12	<.01	−0.31 to0.15
IOLMaster-EyeSys	0.32±0.12	<.01	0.07 to 0.56
IOLMaster-Medmont	−0.08±0.15	<.01	−0.37 to 0.21
IOLMaster-Topolyzer	0.07±0.08	<.01	−0.082 to 0.219
IOLMaster-Pentacam	0.07±0.11	<.01	−0.16 to 0.29
IOLMaster-Sirius	0.00±0.11	.859	−0.22 to 0.22
EyeSys-Medmont	−0.40±0.17	<.01	−0.73 to −0.06
EyeSys-Topolyzer	−0.25±0.13	<.01	−0.5 to 0
EyeSys-Pentacam	−0.25±0.11	<.01	−0.47 to 0.03
EyeSys-Sirius	−0.32±0.12	<.01	−0.56 to −0.08
Medmont-Topolyzer	0.15±0.15	<.01	−0.14 to 0.44
Medmont-Pentacam	0.14±0.14	<.01	−0.14 to 0.43
Medmont-Sirius	0.08±0.17	.014	−0.26 to 0.42
Topolyzer-Pentacam	0.00±0.11	.872	−0.23 to 0.22
Topolyzer-Sirius	−0.07±0.12	<.01	−0.31 to0.17
Pentacam-Sirius	−0.07±0.10	<.01	−0.263 to 0.126

D = diopter, SD = standard deviation, LoA = limits of agreement.

**Table 5 pone-0045607-t005:** Comparison of the Steep Keratometry between 8 Different Devices.

Devices	Mean Difference(D) ± SD	*P* Value	95% LoA (D)
Tomey-Topcon	0.13±0.12	<.01	−0.100 to 0.360
Tomey-IOLMaster	−0.10±0.13	<.01	−0.360 to 0.160
Tomey-EyeSys	0.34±0.14	<.01	0.060 to 0.620
Tomey-Medmont	−0.21±0.18	<.01	−0.560 to 0.140
Tomey-Topolyzer	0.03±0.18	.346	−0.330 to 0.390
Tomey-Pentacam	0.02±0.14	.357	−0.260 to 0.310
Tomey-Sirius	−0.03±0.14	.220	−0.310 to 0.25
Topcon-IOLMaster	−0.23±0.13	<.01	−0.48 to 0.02
Topcon-EyeSys	0.21±0.17	<.01	−0.12 to 0.53
Topcon-Medmont	−0.34±0.18	<.01	−0.69 to 0
Topcon-Topolyzer	−0.07±0.06	<.01	−0.41 to 0.21
Topcon-Pentacam	−0.11±0.16	<.01	−0.42 to 0.2
Topcon-Sirius	−0.16±0.14	<.01	−0.43 to 0.11
IOLMaster-EyeSys	0.44±0.17	<.01	0.11 to 0.77
IOLMaster-Medmont	−0.11±0.22	<.01	−0.54 to 0.32
IOLMaster-Topolyzer	0.13±0.21	<.01	−0.28 to 0.54
IOLMaster-Pentacam	0.12±0.13	<.01	−0.13 to 0.37
IOLMaster-Sirius	0.07±0.17	.021	−0.27 to 0.41
EyeSys-Medmont	−0.55±0.17	<.01	−0.88 to −0.22
EyeSys-Topolyzer	−0.31±0.19	<.01	−0.68 to 0.06
EyeSys-Pentacam	−0.32±0.14	<.01	−0.60 to −0.03
EyeSys-Sirius	−0.37±0.16	<.01	−0.68 to −0.06
Medmont-Topolyzer	0.24±0.17	<.01	−0.09 to 0.57
Medmont-Pentacam	0.23±0.18	<.01	−0.12 to 0.59
Medmont-Sirius	0.18±0.18	<.01	−0.18 to 0.54
Topolyzer-Pentacam	−0.01±0.19	.838	−0.38 to 0.36
Topolyzer-Sirius	−0.06±0.15	.025	−0.36 to 0.24
Pentacam-Sirius	−0.05±0.16	.053	−0.36 to 0.25

D = diopter, SD = standard deviation, LoA = limits of agreement.

**Table 6:** Comparison of Mean Keratometry between 8 Different Devices.

Devices	Mean Difference(D) ± SD	*P* Value	95% LoA (D)
Tomey-Topcon	0.04±0.08	<.01	−0.11 to 0.20
Tomey-IOLMaster	−0.11±0.08	<.01	−0.268 to 0.05
Tomey-EyeSys	0.27±0.11	<.01	0.06 to 0.48
Tomey-Medmont	−0.21±0.12	<.01	−0.45 to 0.04
Tomey-Topolyzer	−0.01±0.11	.694	−0.22 to 0.21
Tomey-Pentacam	−0.01±0.10	.439	−0.22 to 0.19
Tomey-Sirius	−0.08±0.10	<.01	−0.26 to 0.11
Topcon-IOLMaster	−0.15±0.08	<.01	−0.317 to 0.11
Topcon-EyeSys	0.23±0.14	<.01	−0.04 to 0.5
Topcon-Medmont	−0.25±0.13	<.01	−0.51to 0.01
Topcon-Topolyzer	−0.10±0.16	<.01	−0.25 to 0.15
Topcon-Pentacam	−0.06±0.12	<.01	−0.29 to 0.18
Topcon-Sirius	−0.12±0.11	<.01	−0.33 to 0.09
IOLMaster-EyeSys	0.38±0.12	<.01	0.14 to 0.62
IOLMaster-Medmont	−0.10±0.15	<.01	−0.39 to 0.20
IOLMaster-Topolyzer	0.10±0.11	<.01	−0.12 to 0.32
IOLMaster-Pentacam	0.10±0.10	<.01	−0.091 to 0.283
IOLMaster-Sirius	0.03±0.11	.074	−0.18 to 0.25
EyeSys-Medmont	−0.48±0.15	<.01	−0.77 to −0.18
EyeSys-Topolyzer	−0.28±0.14	<.01	−0.5 5to 0
EyeSys-Pentacam	−0.28±0.11	<.01	−0.49 to −0.07
EyeSys-Sirius	−0.35±0.13	<.01	−0.6 to −0.09
Medmont-Topolyzer	0.19±0.14	<.01	−0.09 to 0.47
Medmont-Pentacam	0.20±0.14	<.01	−0.08 to 0.47
Medmont-Sirius	0.13±0.16	<.01	−0.18 to 0.45
Topolyzer-Pentacam	−0.01±0.14	.793	−0.28 to 0.27
Topolyzer-Sirius	−0.07±0.12	<.01	−0.31 to 0.18
Pentacam-Sirius	−0.06±0.11	<.01	−0.28 to 0.16

D = diopter, SD = standard deviation, LoA = limits of agreement.

There were statistically significant differences in Kf between any two paired devices except for Topcon-Topolyzer, Topcon-Pentacam, IOLMaster-Sirius, and Topolyzer-Pentacam comparisons (Table 4). For Ks, there were insignificant differences between the Tomey and Topolyzer, Tomey and Pentacam, Tomey and Sirius, Topolyzer and Pentacam, and Pentacam and Sirius. As shown in Tables 4 to [Table pone-0045607-t005] 6, the Kf, Ks and Kave mean values of the Medmont were the largest, while the Kf, Ks and Kave readings obtained by the EyeSys were the smallest.

As regards Kave (table 6), Tomey and Topolyzer, Tomey and Pentacam, IOLMaster and Sirius, and Topolyzer and Pentacam were not significantly different. Tables S8 and S9 showed significant differences in both J_0_ and J_45_: for the former such differences were limited to the Tomey, Topcon and IOLMaster devices, for the latter they interested all instruments but the EyeSys.

As regards agreement, Table 4 shows that the 95% LoAs for Kf were <0.5 D when comparing all pairs of instruments. The only exception being the EyeSys and Medmont corneal topographers, whose agreement with respect to other devices was lower.

Agreement among the 8 devices was lower for Ks, as shown in Table 5: the 95% LoAs were equal or smaller than 0.5D for almost instruments, except for EyeSys and Medmont. Among these paired comparisons, the largest 95% LoA were obtained for the EyeSys-Medmont comparison (−0.88 to −0.22 D).

In the Bland-Altman analysis of Kave (Table 6), the 95% LoA were equal or larger than 0.5D when evaluating the Medmont and EyeSys corneal topographers and lower than 0.5D for other comparisons. For the 95% LoAs of J_0_ and J_45_, they were all ≤ ±0.31 among any two paired devices.

## Discussion

Accurate measurements of corneal power and astigmatism represent a crucial need requirement in this era of refractive cataract surgery: the former is needed by all formulas calculating IOL power, the latter is needed when planning toric IOL implantation or surgical correction of astigmatism by excimer laser. In this prospective study, we assessed the intrasession repeatability, intersession reproducibility, and agreement of corneal powers obtained from 8 different devices. To our knowledge, no previous study has assessed the precision and interchangeability of keratometry on such a large number of instruments. Moreover, while several studies have assessed the repeatability of one ore more instruments in measuring corneal power, in only a very few cases has the repeatability of astigmatism measurements been carried out by means of vector analysis.

All devices demonstrated excellent intrasession repeatability and intersession reproducibility in measuring Kf and Ks and Kave (ICC ≥0.98 for all). The vector power Ms were as the SimK. The repeatability and reproducibility of vectors J_0_ and J_45_ was slightly lower, but still reasonably good. As regards repeatability, the ICC of J_0_ and J_45_ ranged respectively from 0.925 (EyeSys) to 0.994 (Tomey and IOLMaster) and from 0.747 (Medmont) to 0.982 (Tomey). When evaluating the reproducibility, the ICC of J_0_ and J_45_ ranged, respectively, from 0.917(Medmont) to 0.990 (Topolyzer) and from 0.803 (Medmont) to 0.971 (Topolyzer).

In comparing the 8 devices, the means of the differences were similar, which suggests a good degree of concordance among them. However in some cases (EyeSys and Medmont) agreement was only fair, so that caution is recommended when using some of them interchangeably.

### IOLMaster

Our data confirm the excellent intrasession repeatability of corneal power measurements by the IOLMaster, as previously reported by other authors. [7], [10] Like in the study by Shirayama et al., [7] the IOLMaster showed the lowest COV in comparison to the other devices tested. We also observed a good intersession reproducibility of the keratometry by IOLMaster, with ICCs for Kf and Ks even higher than that reported by Shammas et al. [29].

Previous studies found that the IOLMaster provided slightly steeper corneal power readings than manual keratometry, automated keratometry, Placido disc corneal topography, Scheimpflug imaging, and Scheimpflug imaging combined with Placido disc corneal topography. [7], [11], [30] This is in good agreement with our findings, as we observed that IOLMaster corneal power measurements were higher than those provided by all instruments except the Medmont. Steeper corneal power values by the IOLMaster may be related to the more central corneal curvature readings of this instrument, which takes measurements over a diameter of approximately 2.5 mm. It is known that the central corneal curvature is steeper than the peripheral curvature in a normal prolate cornea. [7], [11], [12].

The IOLMaster also offered high repeatability for astigmatic vector analysis as well as good, although slightly lower, reproducibility. To our knowledge, this is the first study to evaluate repeatability and reproducibility of J_0_ and J_45_ measurements by the IOLMaster.

### Tomey RC-5000 and Topcon KR-8000

In this study both autokeratometers offered excellent repeatability and reproducibility, thus confirming the data previously reported in children by Huynh et al. [11] for another Auto-Keratometer (RK-F1, Canon, Japan) based on the same principle. In the present study, even higher degrees of reliability were found for the measurement of Kf and Ks by RC-5000 and KR-8000, suggesting that keratometry measurements in adults were more repeatable than those in children, perhaps because children have difficulty in maintaining proper posture during the image-acquiring procedures and have a short attention span.

Repeatability of both autokeratometers was slightly higher than that of the EyeSys corneal topography, a result that had already been reported for a previous version of the Topcon autokeratometer. [31].

Repeatability and reproducibility of astigmatic vectors were high.

### Medmont E300

The E300 has already been found to be a highly accurate and repeatable corneal topographer. [5], [22] Our results are in good agreement with previous investigations for corneal power measurements. On the contrary, the repeatability and reproducibility of the E300 were lower (compared to other instruments in this study) for J_45_.

The E300 produced the highest Kf, Ks and Kave among the whole set of instruments. This is consistent with previous studies demonstrating that the E300 gives significantly steeper corneal power values than other devices. [4], [13] As a consequence, agreement with instruments providing the lowest Kf and Ks (such as EyeSys) was moderate.

### EyeSys Vista

In this study the EyeSys topographer showed good repeatability and reproducibility of corneal curvature measurements, although the results were slightly lower than those of most instruments. High repeatability of the EyeSys for corneal curvature measurements had already been reported. [14], [15].

Agreement between EyeSys, which provided the flattest keratometry values, and the other instruments was fair. The 95% LoAs between EyeSys and the other 7 devices were all larger than ±0.50 D. Clearly, this range does not allow this instrument to be used interchangeably with other devices. These results are consistent with the findings of previous studies. Stefano et al. [32] compared the keratometry values obtained by EyeSys with those obtained by the Pentacam. Although they found a high correlation between the measurements obtained with both devices, the 95% LoA range from −1.06 to 1.26 D and −0.87 to 0.85 D for Kf and Ks, respectively, suggests that these limits were too large to consider both instruments are interchangeable. Similarly, Tsilimbaris et al. [33] compared the measurements obtained using the EyeSys and the Javal keratometer and found that there was no significant difference between the instruments. However, the 95% LoA ranged from −0.87 to 0.93 D, which also showed that the two instruments were in poor agreement. Other studies have also reported similar findings when measurements were taken with manual keratometry and Placido-based topography. [16], [17] These devices were in poor agreement, although a good correlation between different keratometric methods was observed. [28].

### Topolyzer

To date, no study has reported on the precision of keratometry measurements obtained by the Topolyzer, thought it was an effective and safe tool in topography-guided corneal excimer laser surgery to correct myopia, hyperopia, and mixed astigmatism. [34], [35] The same model marketed by Oculus (Keratograph, Oculus, Germany) has been used to assess corneal wavefront aberrations. [36] Our results represent the first confirmation that the Topolyzer displays excellent reliability in measuring corneal power (ICCs ≥0.971) and astigmatism (ICCs >0.97 for both J_0_ and J_45_).

The current study is also the first to demonstrate good agreement between the Topolyzer and other devices. The 95% LoA values of K were lower than 0.5 D in most cases, except in the case of the EyeSys Vista or Medmont E3000.

### Sirius

According to our data, measurements by the Scheimpflug camera combined with Placido corneal topography (Sirius) showed good repeatability and reproducibility. Results for keratometry are quite similar to those previously reported for intrasession repeatability of SimK by the same device. [6] Results for astigmatism decomposition components had never been reported.

Sirius is a relatively new instrument and only a few studies have evaluated its ability to measure corneal curvature and power. [18] Savini et al. [18] compared the anterior segment measurements provided by 3 Scheimpflug topographers and 1 Placido corneal topographer in 25 subjects. Although the mean SimK was significantly different among the 4 instruments, post-test analysis did not reveal any statistically significant difference between Pentacam and Sirius. In good agreement, our study did not find a statistically significant difference between the two instruments for the steep K and a statistically but not clinically significant difference for the flat K. The 95% LoA between Pentacam and Sirius were slightly larger in the study by Savini et al. (−0.59 to 0.59 D) than in ours (−0.28 to 0.16 D). This discrepancy may be related to the different age of the two samples, as the mean age of Savini’s sample (age 57.9 years ±21.2) was higher than the mean age of our sample: young subjects have better fixation, and stability of the tear film than older patients.

### Pentacam

The Pentacam offered high repeatability and reproducibility in measuring corneal curvature, thus confirming the findings of previous studies that investigated its repeatability and agreement with respect to other instruments. [5], [9], [19] The values recently reported by McAlinden et al. [19] for Kf and Ks, as provided by the Pentacam HR, and those described by Shankar et al., [20] using the original Pentacam (not HR), are very close to ours. A comparison for astigmatism is difficult as both studies did not evaluate vector analysis. [19], [20] The latter was carried out by Read et al., [5] who reported repeatability values similar to ours, although in their study the performance of Pentacam was slightly worse than that of the Medmont, whereas in our sample the opposite was true.

Comparison of the Pentacam to other instruments showed good agreement (LoA <0.5D) and little mean differences in Kf and Ks in most cases. Recently, several authors compared corneal curvature measurements by the Pentacam to those of other instruments. In 2011 Savini et al. [18] reported no difference in the mean corneal power (SimK) of the Pentacam and Sirius (see above). In 2009 the same authors did not find any statistically significant difference among the Pentacam and two Placido disc corneal topographers, but the 95% LoA were large enough to be considered clinically significant. [37].

### Limitations

There are several limitations of the present study. First, the results are based on a relatively small number of eyes, although this number is equal to or higher than those used in previous studies. [4], [7], [8], [10], [14], [15], [17], [22] Second, our study is limited to young and healthy subjects with normal corneas and good fixation; the understanding and collaboration of these subjects are very good; and keratometry scans images were of excellent quality. In older patients with corneal abnormalities or subjected to post-laser in situ keratomileusis or corneal surface ablation surgery, the results may be different and could include additional variability. Further research is required to comprehensively assess the validity and precision of the corneal power measurements obtained by different keratometric devices in such cases. Third, our study is limited to the intraobserver repeatability and intersession reproducibility of corneal power measurements by these devices. The variability of the measurement system caused by different observers deserves further investigation. Finally, from a practical point of view, although some instruments showed no statistically significant inter-device differences and good agreement and the result may suggest their measurements can be used interchangeably in IOL power calculation, we still suggest optimizing the constants of IOL power calculation formulas when changing from one instrument to another. More studies are needed to report these constants.

### Conclusion

In summary, our data showed that anterior corneal curvature measurements obtained from 8 different devices present very good repeatability and reproducibility. The results obtained using the Tomey RC-5000, Topcon KR-8000, IOLMaster, Allegro Topolyzer, Pentacam and Sirius were well correlated and comparable, suggesting that they could be used interchangeably in most clinical settings. However, caution is warranted when using measurements obtained by the EyeSys Vista and the Medmont. It is inadvisable to use both devices interchangeably with other devices in every clinical situation.

## Supporting Information

Table S1 **Intrasession Repeatability of 8 Different Devices in Measuring vector J_0_ (N = 35).**
(DOCX)

Table S2 **Intrasession Repeatability of 8 Different Devices in Measuring vector J_45_ (N = 35).**
(DOCX)

Table S3 **Intersession Reproducibility of 8 Different Devices in Measuring Flat Keratometry (N = 35).**
(DOCX)

Table S4 **Intersession Reproducibility of 8 Different Devices in Measuring Steep Keratometry (N = 35).**
(DOCX)

Table S5 **Intersession Reproducibility of 8 Different Devices in Measuring Mean Keratometry (N = 35).**
(DOCX)

Table S6 **Intersession Reproducibility of 8 Different Devices in Measuring vector J_0_ (N = 35).**
(DOCX)

Table S7 **Intersession Reproducibility of 8 Different Devices in Measuring vector J_45_ (N = 35).**
(DOCX)

Table S8 **Comparison of the vector J_0_ between 8 Different Devices.**
(DOCX)

Table S9 **Comparison of the vector J_45_ between 8 Different Devices.**
(DOCX)

Checklist S1 **CONSORT Checklist.**
(DOC)

Protocol S1 **Trial Protocol.**
(DOC)

## References

[1] Lee AC, Qazi MA, Pepose JS (2008) Biometry and intraocular lens power calculation. Curr Opin Ophthalmol 19: 13–17. 18090891 10.1097/ICU.0b013e3282f1c5ad

[2] Tomidokoro A, Oshika T, Amano S, Higaki S, Maeda N, et al (2000) Changes in anterior and posterior corneal curvatures in keratoconus. Ophthalmology 107: 1328–1332. 10889107 10.1016/s0161-6420(00)00159-7

[3] Jinabhai A, Radhakrishnan H, O'Donnell C (2011) Pellucid corneal marginal degeneration: A review. Cont Lens Anterior Eye 34: 56–63. 21185225 10.1016/j.clae.2010.11.007

[4] Chui WS, Cho P (2005) A comparative study of the performance of different corneal topographers on children with respect to orthokeratology practice. Optom Vis Sci 82: 420–427. 15894918 10.1097/01.OPX.0000162642.24885.71

[5] Read SA, Collins MJ, Iskander DR, Davis BA (2009) Corneal topography with Scheimpflug imaging and videokeratography: comparative study of normal eyes. J Cataract Refract Surg 35: 1072–1081. 19465294 10.1016/j.jcrs.2009.01.020

[6] Savini G, Barboni P, Carbonelli M, Hoffer KJ (2011) Repeatability of automatic measurements by a new Scheimpflug camera combined with Placido topography. J Cataract Refract Surg 37: 1809–1816. 21852068 10.1016/j.jcrs.2011.04.033

[7] Shirayama M, Wang L, Weikert MP, Koch DD (2009) Comparison of corneal powers obtained from 4 different devices. Am J Ophthalmol 148: 528–535 e521. 10.1016/j.ajo.2009.04.028 19541287

[8] Wang L, Shirayama M, Koch DD (2010) Repeatability of corneal power and wavefront aberration measurements with a dual-Scheimpflug Placido corneal topographer. J Cataract Refract Surg 36: 425–430. 20202540 10.1016/j.jcrs.2009.09.034

[9] Chen D, Lam AK (2009) Reliability and repeatability of the Pentacam on corneal curvatures. Clin Exp Optom 92: 110–118. 18983633 10.1111/j.1444-0938.2008.00336.x

[10] Vogel A, Dick HB, Krummenauer F (2001) Reproducibility of optical biometry using partial coherence interferometry : intraobserver and interobserver reliability. J Cataract Refract Surg 27: 1961–1968. 11738911 10.1016/s0886-3350(01)01214-7

[11] Huynh SC, Mai TQ, Kifley A, Wang JJ, Rose KA, et al (2006) An evaluation of keratometry in 6-year-old children. Cornea 25: 383–387. 16670473 10.1097/01.ico.0000214203.84081.ec

[12] Huang J, Pesudovs K, Wen D, Chen S, Wright T, et al (2011) Comparison of anterior segment measurements with rotating Scheimpflug photography and partial coherence reflectometry. J Cataract Refract Surg 37: 341–348. 21241919 10.1016/j.jcrs.2010.08.044

[13] Gonzalez-Meijome JM, Jorge J, Queiros A, Almeida JB, Parafita MA (2004) A comparison of the ARK-700A autokeratometer and Medmont E300 corneal topographer when measuring peripheral corneal curvature. Ophthalmic Physiol Opt 24: 391–398. 15315653 10.1111/j.1475-1313.2004.00203.x

[14] Gonzalez Perez J, Cervino A, Giraldez MJ, Parafita M, Yebra-Pimentel E (2004) Accuracy and precision of EyeSys and Orbscan systems on calibrated spherical test surfaces. Eye Contact Lens 30: 74–78. 15260351 10.1097/01.icl.0000111749.04644.92

[15] Jeandervin M, Barr J (1998) Comparison of repeat videokeratography: repeatability and accuracy. Optom Vis Sci 75: 663–669. 9778699 10.1097/00006324-199809000-00021

[16] Varssano D, Rapuano CJ, Luchs JI (1997) Comparison of keratometric values of healthy and diseased eyes measured by Javal keratometer, EyeSys, and PAR. J Cataract Refract Surg 23: 419–422. 9159687 10.1016/s0886-3350(97)80187-3

[17] Dave T, Ruston D, Fowler C (1998) Evaluation of the EyeSys model II computerized videokeratoscope. Part I: Clinical assessment. Optom Vis Sci 75: 647–655. 9778697 10.1097/00006324-199809000-00019

[18] Savini G, Carbonelli M, Sbreglia A, Barboni P, Deluigi G, et al (2011) Comparison of anterior segment measurements by 3 Scheimpflug tomographers and 1 Placido corneal topographer. J Cataract Refract Surg 37: 1679–1685. 21855765 10.1016/j.jcrs.2011.03.055

[19] McAlinden C, Khadka J, Pesudovs K (2011) A Comprehensive Evaluation of the Precision (Repeatability and Reproducibility) of the Oculus Pentacam HR. Invest Ophthalmol Vis Sci 52: 7731–7737. 21810981 10.1167/iovs.10-7093

[20] Shankar H, Taranath D, Santhirathelagan CT, Pesudovs K (2008) Anterior segment biometry with the Pentacam: comprehensive assessment of repeatability of automated measurements. J Cataract Refract Surg 34: 103–113. 18165089 10.1016/j.jcrs.2007.09.013

[21] Tang M, Chen A, Li Y, Huang D (2010) Corneal power measurement with Fourier-domain optical coherence tomography. J Cataract Refract Surg 36: 2115–2122. 21111315 10.1016/j.jcrs.2010.07.018 PMC3005697

[22] Cho P, Lam AK, Mountford J, Ng L (2002) The performance of four different corneal topographers on normal human corneas and its impact on orthokeratology lens fitting. Optom Vis Sci 79: 175–183. 11915858 10.1097/00006324-200203000-00012

[23] British Standards Institution (1994) Accuracy (Trueness and Precision) of Measurement Methods and Results: General Principles and Definitions. London: HMO BS ISO 5725 part 1.

[24] British Standards Institution (1994) Accuracy (Trueness and Precision) of Measurement Methods and Results: Basic Methods for the Determination of Repeatability and Reproducibility of a Standard Measurement Method. London: HMO BS ISO 5725 part 2.

[25] Bland JM, Altman DG (1986) Statistical methods for assessing agreement between two methods of clinical measurement. Lancet 8: 307–310. 2868172

[26] Collins MJ, Buehren T, Bece A, Voetz SC (2006) Corneal optics after reading, microscopy and computer work. Acta Ophthalmol Scand 84: 216–224. 16637840 10.1111/j.1600-0420.2005.00547.x

[27] Thibos LN, Wheeler W, Horner D (1997) Power vectors: an application of Fourier analysis to the description and statistical analysis of refractive error. Optom Vis Sci 74: 367–375. 9255814 10.1097/00006324-199706000-00019

[28] Bland JM, Altman DG (1996) Measurement error. BMJ 313: 744. 8819450 10.1136/bmj.313.7059.744 PMC2352101

[29] Shammas HJ, Chan S (2010) Precision of biometry, keratometry, and refractive measurements with a partial coherence interferometry-keratometry device. J Cataract Refract Surg 36: 1474–1478. 20692557 10.1016/j.jcrs.2010.02.027

[30] Savini G, Barboni P, Carbonelli M, Hoffer KJ (2009) Accuracy of Scheimpflug corneal power measurements for intraocular lens power calculation. J Cataract Refract Surg 35: 1193–1197. 19545807 10.1016/j.jcrs.2009.02.031

[31] Pardhan S, Douthwaite WA (1998) Comparison of videokeratoscope and autokeratometer measurements on ellipsoid surfaces and human corneas. J Refract Surg 14: 414–419. 9699165 10.3928/1081-597X-19980701-07

[32] Stefano VS, Melo Junior LA, Mallmann F, Schor P (2010) Interchangeability between Placido disc and Scheimpflug system: quantitative and qualitative analysis. Arq Bras Oftalmol 73: 363–366. 20944942 10.1590/s0004-27492010000400013

[33] Tsilimbaris MK, Vlachonikolis IG, Siganos D, Makridakis G, Pallikaris IG (1991) Comparison of keratometric readings as obtained by Javal Ophthalmometer and Corneal Analysis System (EyeSys). Refract Corneal Surg 7: 368–373. 1958623

[34] Cummings AB, Mascharka N (2010) Outcomes after topography-based LASIK and LASEK with the wavelight oculyzer and topolyzer platforms. J Refract Surg 26: 478–485. 19715264 10.3928/1081597X-20090814-05

[35] Falavarjani KG, Hashemi M, Modarres M, Sanjari MS, Darvish N, et al (2011) Topography-guided vs wavefront-optimized surface ablation for myopia using the WaveLight platform: a contralateral eye study. J Refract Surg 27: 13–17. 20349856 10.3928/1081597X-20100310-02

[36] Iseli HP, Jankov M, Bueeler M, Wimmersberger Y, Seiler T, et al (2006) Corneal and total wavefront aberrations in phakic and pseudophakic eyes after implantation of monofocal foldable intraocular lenses. J Cataract Refract Surg 32: 762–771. 16765792 10.1016/j.jcrs.2005.10.032

[37] Savini G, Barboni P, Carbonelli M, Hoffer KJ (2009) Agreement between Pentacam and videokeratography in corneal power assessment. J Refract Surg 25: 534–538. 19603621 10.3928/1081597X-20090512-07

