# Supplementary material for: A Comprehensive Assessment of the Precision and Agreement of Anterior Corneal Power Measurements Obtained Using 8 Different Devices
Source: PLoS One. 2012 Sep 25;7(9):e45607. doi: 10.1371/journal.pone.0045607 (PMC3458095; doi:10.1371/journal.pone.0045607)
Supplement: Table S1 — Intrasession Repeatability of 8 Different Devices in Measuring vector J0 (N = 35). (DOCX) [file pone.0045607.s001.docx]

| Table S1. Intrasession Repeatability of 8 Different Devices in Measuring vector J_0_ (N = 35) | | | | | |
| --- | --- | --- | --- | --- | --- |
| Device | Session | Mean ± SD | Sw | 2.77 Sw | ICC |
| Tomey RC | 1st | 0.43 ± 0.37 | 0.03 | 0.09 | 0.992 |
|  | 2nd | 0.42 ± 0.37 | 0.03 | 0.08 | 0.994 |
| Topcon KR | 1st | 0.32 ± 0.33 | 0.04 | 0.10 | 0.987 |
|  | 2nd | 0.33 ± 0.32 | 0.04 | 0.11 | 0.985 |
| IOLMaster | 1st | 0.39 ± 0.39 | 0.03 | 0.09 | 0.994 |
|  | 2nd | 0.39 ± 0.37 | 0.04 | 0.12 | 0.987 |
| EyeSys Vista | 1st | 0.37 ± 0.35 | 0.06 | 0.16 | 0.974 |
|  | 2nd | 0.38 ± 0.34 | 0.10 | 0.27 | 0.925 |
| Medmont | 1st | 0.38 ± 0.40 | 0.07 | 0.19 | 0.971 |
|  | 2nd | 0.42 ± 0.39 | 0.06 | 0.16 | 0.978 |
| Topolyzer | 1st | 0.39 ± 0.36 | 0.04 | 0.08 | 0.988 |
|  | 2nd | 0.40 ± 0.34 | 0.04 | 0.11 | 0.988 |
| Pentacam | 1st | 0.39 ± 0.37 | 0.06 | 0.15 | 0.978 |
|  | 2nd | 0.41 ± 0.36 | 0.06 | 0.17 | 0.973 |
| Sirius | 1st | 0.38 ± 0.36 | 0.06 | 0.17 | 0.972 |
|  | 2nd | 0.41 ± 0.35 | 0.04 | 0.12 | 0.985 |
| SD = standard deviation, Sw = within-subject standard deviation, ICC = intraclass correlation coefficient. | | | | | |
