# Supplementary material for: A Comprehensive Assessment of the Precision and Agreement of Anterior Corneal Power Measurements Obtained Using 8 Different Devices
Source: PLoS One. 2012 Sep 25;7(9):e45607. doi: 10.1371/journal.pone.0045607 (PMC3458095; doi:10.1371/journal.pone.0045607)
Supplement: Table S2 — Intrasession Repeatability of 8 Different Devices in Measuring vector J45 (N = 35). (DOCX) [file pone.0045607.s002.docx]

| Table S2. Intrasession Repeatability of 8 Different Devices in Measuring vector J_45_ (N = 35) | | | | | |
| --- | --- | --- | --- | --- | --- |
| Device | Session | Mean ± SD | Sw | 2.77 Sw | ICC |
| Tomey RC | 1st | -0.11 ± 0.17 | 0.04 | 0.10 | 0.955 |
|  | 2nd | -0.09 ± 0.18 | 0.02 | 0.07 | 0.982 |
| Topcon KR | 1st | -0.12 ± 0.16 | 0.03 | 0.08 | 0.969 |
|  | 2nd | -0.11 ± 0.17 | 0.03 | 0.09 | 0.968 |
| IOLMaster | 1st | -0.10 ± 0.19 | 0.03 | 0.09 | 0.971 |
|  | 2nd | -0.09 ± 0.20 | 0.03 | 0.09 | 0.973 |
| EyeSys Vista | 1st | -0.03 ± 0.16 | 0.03 | 0.09 | 0.956 |
|  | 2nd | -0.02 ± 0.16 | 0.04 | 0.12 | 0.929 |
| Medmont | 1st | -0.04 ± 0.23 | 0.13 | 0.35 | 0.747 |
|  | 2nd | -0.05 ± 0.25 | 0.10 | 0.28 | 0.844 |
| Topolyzer | 1st | -0.05 ± 0.17 | 0.03 | 0.08 | 0.972 |
|  | 2nd | -0.04 ± 0.17 | 0.03 | 0.07 | 0.975 |
| Pentacam | 1st | -0.01 ± 0.17 | 0.07 | 0.19 | 0.850 |
|  | 2nd | -0.02 ± 0.17 | 0.07 | 0.18 | 0.860 |
| Sirius | 1st | -0.07 ± 0.17 | 0.04 | 0.12 | 0.940 |
|  | 2nd | -0.07 ± 0.17 | 0.03 | 0.09 | 0.967 |
| SD = standard deviation, Sw = within-subject standard deviation, ICC = intraclass correlation coefficient. | | | | | |
