# Supplementary material for: A Comprehensive Assessment of the Precision and Agreement of Anterior Corneal Power Measurements Obtained Using 8 Different Devices
Source: PLoS One. 2012 Sep 25;7(9):e45607. doi: 10.1371/journal.pone.0045607 (PMC3458095; doi:10.1371/journal.pone.0045607)
Supplement: Table S4 — Intersession Reproducibility of 8 Different Devices in Measuring Steep Keratometry (N = 35). (DOCX) [file pone.0045607.s004.docx]

| Table S4. Intersession Reproducibility of 8 Different Devices in Measuring Steep Keratometry (N = 35) | | | | | |
| --- | --- | --- | --- | --- | --- |
| Device | Mean difference (D) ± SD | Sw (D) | 2.77 Sw (D) | COV (%) | ICC |
| Tomey RC | 0.02 ± 0.14 | 0.10 | 0.27 | 0.22 | 0.995 |
| Topcon KR | -0.02 ± 0.13 | 0.09 | 0.26 | 0.21 | 0.996 |
| IOLMaster | 0.01 ± 0.14 | 0.10 | 0.28 | 0.18 | 0.995 |
| EyeSys Vista | -0.01 ± 0.16 | 0.11 | 0.26 | 0.26 | 0.993 |
| Medmont | -0.06 ± 0.17 | 0.13 | 0.35 | 0.28 | 0.993 |
| Topolyzer | 0.00 ± 0.12 | 0.08 | 0.23 | 0.19 | 0.997 |
| Pentacam | -0.01 ± 0.10 | 0.07 | 0.18 | 0.15 | 0.998 |
| Sirius | -0.05 ± 0.14 | 0.10 | 0.29 | 0.23 | 0.995 |
| D = diopter, SD = standard deviation, Sw = within-subject standard deviation, COV = within-subject coefficient of variation, ICC = intraclass correlation coefficient. | | | | | |
