# Supplementary material for: A Comprehensive Assessment of the Precision and Agreement of Anterior Corneal Power Measurements Obtained Using 8 Different Devices
Source: PLoS One. 2012 Sep 25;7(9):e45607. doi: 10.1371/journal.pone.0045607 (PMC3458095; doi:10.1371/journal.pone.0045607)
Supplement: Table S6 — Intersession Reproducibility of 8 Different Devices in Measuring vector J0 (N = 35). (DOCX) [file pone.0045607.s006.docx]

| Table S6. Intersession Reproducibility of 8 Different Devices in Measuring vector J_0_ (N = 35) | | | | |
| --- | --- | --- | --- | --- |
| Device | Mean difference ± SD | Sw | 2.77 Sw | ICC |
| Tomey RC | 0.00 ± 0.08 | 0.06 | 0.15 | 0.977 |
| Topcon KR | -0.01 ± 0.06 | 0.04 | 0.12 | 0.982 |
| IOLMaster | 0.00 ± 0.08 | 0.06 | 0.16 | 0.978 |
| EyeSys Vista | -0.01 ± 0.06 | 0.05 | 0.13 | 0.983 |
| Medmont | -0.04 ± 0.10 | 0.07 | 0.22 | 0.917 |
| Topolyzer | -0.01 ± 0.05 | 0.04 | 0.10 | 0.990 |
| Pentacam | -0.01 ± 0.06 | 0.04 | 0.12 | 0.986 |
| Sirius | -0.03 ± 0.06 | 0.05 | 0.13 | 0.983 |
| SD = standard deviation, Sw = within-subject standard deviation, ICC = intraclass correlation coefficient. | | | | |
