# Supplementary material for: A Comprehensive Assessment of the Precision and Agreement of Anterior Corneal Power Measurements Obtained Using 8 Different Devices
Source: PLoS One. 2012 Sep 25;7(9):e45607. doi: 10.1371/journal.pone.0045607 (PMC3458095; doi:10.1371/journal.pone.0045607)
Supplement: Table S7 — Intersession Reproducibility of 8 Different Devices in Measuring vector J45 (N = 35). (DOCX) [file pone.0045607.s007.docx]

| Table S7. Intersession Reproducibility of 8 Different Devices in Measuring vector J_45_ (N = 35) | | | | |  |
| --- | --- | --- | --- | --- | --- |
| Device | Mean difference ± SD | Sw | 2.77 Sw | ICC | |
| Tomey RC | -0.02 ± 0.07 | 0.05 | 0.14 | 0.922 | |
| Topcon KR | -0.01 ± 0.04 | 0.03 | 0.09 | 0.965 | |
| IOLMaster | -0.01 ± 0.09 | 0.06 | 0.17 | 0.898 | |
| EyeSys Vista | -0.01 ± 0.04 | 0.03 | 0.08 | 0.964 | |
| Medmont | 0.01 ± 0.15 | 0.11 | 0.29 | 0.803 | |
| Topolyzer | -0.01 ± 0.04 | 0.03 | 0.06 | 0.971 | |
| Pentacam | 0.01 ± 0.07 | 0.05 | 0.14 | 0.906 | |
| Sirius | -0.01 ± 0.05 | 0.04 | 0.10 | 0.953 | |
| D = diopter, SD = standard deviation, Sw = within-subject standard deviation, COV = within-subject coefficient of variation, ICC = intraclass correlation coefficient. | | | | |  |
