# Supplementary material for: A Comprehensive Assessment of the Precision and Agreement of Anterior Corneal Power Measurements Obtained Using 8 Different Devices
Source: PLoS One. 2012 Sep 25;7(9):e45607. doi: 10.1371/journal.pone.0045607 (PMC3458095; doi:10.1371/journal.pone.0045607)
Supplement: Table S8 — Comparison of the vector J0 between 8 Different Devices. (DOCX) [file pone.0045607.s008.docx]

| Table S8. Comparison of the vector J_0_ between 8 Different Devices | | | |
| --- | --- | --- | --- |
| Devices | Mean Difference ± SD | *P* Value | 95% LoA |
| Tomey-Topcon | 0.11 ± 0.06 | ＜.01 | -0.014 to 0.228 |
| Tomey-IOLMaster | 0.03 ± 0.07 | ＜.01 | -0.103 to 0.169 |
| Tomey-EyeSys | 0.05 ± 0.07 | ＜.01 | -0.086 to 0.195 |
| Tomey-Medmont | 0.05 ± 0.12 | .019 | -0.18 to 0.29 |
| Tomey-Topolyzer | 0.03 ± 0.09 | .027 | -0.14 to 0.21 |
| Tomey-Pentacam | 0.03 ± 0.07 | .016 | -0.111 to 0.172 |
| Tomey-Sirius | 0.05 ± 0.07 | ＜.01 | -0.089 to 0.191 |
| Topcon-IOLMaster | -0.06 ± 0.07 | ＜.01 | -0.195 to 0.083 |
| Topcon-EyeSys | -0.05 ± 0.07 | ＜.01 | -0.184 to 0.079 |
| Topcon-Medmont | -0.06 ± 0.11 | ＜.01 | -0.27 to 0.16 |
| Topcon-Topolyzer | -0.05 ± 0.10 | ＜.01 | -0.232 to 0.087 |
| Topcon-Pentacam | -0.08 ± 0.07 | ＜.01 | -0.21 to 0.058 |
| Topcon-Sirius | -0.06 ± 0.07 | ＜.01 | -0.195 to 0.083 |
| IOLMaster-EyeSys | 0.02 ± 0.10 | .205 | -0.18 to 0.22 |
| IOLMaster-Medmont | 0.02 ± 0.13 | .466 | -0.23 to 0.26 |
| IOLMaster-Topolyzer | 0.00 ± 0.12 | .920 | -0.23 to 0.23 |
| IOLMaster-Pentacam | 0.00 ± 0.07 | .866 | -0.14 to 0.13 |
| IOLMaster-Sirius | 0.02 ± 0.11 | .330 | -0.2 to 0.23 |
| EyeSys-Medmont | -0.01 ± 0.11 | .726 | -0.21 to 0.2 |
| EyeSys-Topolyzer | -0.02 ± 0.09 | .200 | -0.2 to 0.16 |
| EyeSys-Pentacam | -0.02 ± 0.09 | .105 | -0.19 to 0.14 |
| EyeSys-Sirius | 0.00 ± 0.06 | .710 | -0.12 to 0.11 |
| Medmont-Topolyzer | -0.01 ± 0.10 | .437 | -0.22 to 0.19 |
| Medmont-Pentacam | -0.02 ± 0.11 | .358 | -0.24 to 0.2 |
| Medmont-Sirius | 0.01 ± 0.08 | .856 | -0.17 to 0.18 |
| Topolyzer-Pentacam | 0.00 ± 0.08 | .776 | -0.165 to 0.157 |
| Topolyzer-Sirius | 0.02 ± 0.07 | .162 | -0.116 to 0.148 |
| Pentacam-Sirius | 0.02 ±0.08 | .157 | -0.142 to 0.183 |
| SD = standard deviation, LoA = limits of agreement. | | | |
