# Supplementary material for: A Comprehensive Assessment of the Precision and Agreement of Anterior Corneal Power Measurements Obtained Using 8 Different Devices
Source: PLoS One. 2012 Sep 25;7(9):e45607. doi: 10.1371/journal.pone.0045607 (PMC3458095; doi:10.1371/journal.pone.0045607)
Supplement: Table S9 — Comparison of the vector J45 between 8 Different Devices. (DOCX) [file pone.0045607.s009.docx]

| Table S9. Comparison of the vector J_45_ between 8 Different Devices | | | |  |
| --- | --- | --- | --- | --- |
| Devices | Mean Difference ± SD | *P* Value | 95% LoA | |
| Tomey-Topcon | 0.01 ± 0.05 | .287 | -0.087 to 0.106 | |
| Tomey-IOLMaster | -0.01 ± 0.08 | .350 | -0.163 to 0.138 | |
| Tomey-EyeSys | -0.08 ± 0.07 | ＜.01 | -0.21 to 0.051 | |
| Tomey-Medmont | -0.07 ± 0.11 | ＜.01 | -0.28 to 0.14 | |
| Tomey-Topolyzer | 0.06 ± 0.06 | ＜.01 | -0.169 to 0.052 | |
| Tomey-Pentacam | -0.10 ± 0.08 | ＜.01 | -0.265 to 0.060 | |
| Tomey-Sirius | -0.03 ± 0.05 | ＜.01 | -0.132 to 0.065 | |
| Topcon-IOLMaster | -0.04 ± 0.06 | ＜.01 | -0.154 to 0.068 | |
| Topcon-EyeSys | -0.09 ± 0.08 | ＜.01 | -0.241to 0.063 | |
| Topcon-Medmont | -0.08 ± 0.11 | ＜.01 | -0.3 to 0.14 | |
| Topcon-Topolyzer | -0.07 ± 0.08 | ＜.01 | -0.194 to 0.058 | |
| Topcon-Pentacam | -0.11 ± 0.10 | ＜.01 | -0.31 to 0.09 | |
| Topcon-Sirius | -0.04 ± 0.06 | ＜.01 | -0.154 to 0.068 | |
| IOLMaster-EyeSys | -0.07 ± 0.09 | ＜.01 | -0.241 to 0.107 | |
| IOLMaster-Medmont | -0.06 ± 0.12 | ＜.01 | -0.29 to 0.17 | |
| IOLMaster-Topolyzer | -0.05 ± 0.07 | ＜.01 | -0.188 to 0.096 | |
| IOLMaster-Pentacam | -0.09 ± 0.11 | ＜.01 | -0.31 to 0.13 | |
| IOLMaster-Sirius | -0.02 ± 0.07 | .076 | -0.155 to 0.113 | |
| EyeSys-Medmont | 0.01 ± 0.13 | .730 | -0.24 to 0.26 | |
| EyeSys-Topolyzer | 0.02 ± 0.08 | .123 | -0.13 to 0.18 | |
| EyeSys-Pentacam | -0.02 ± 0.07 | .048 | -0.154 to 0.108 | |
| EyeSys-Sirius | 0.05 ± 0.08 | ＜.01 | -0.1 to 0.19 | |
| Medmont-Topolyzer | 0.01 ± 0.11 | .482 | -0.21 to 0.24 | |
| Medmont-Pentacam | -0.03 ± 0.13 | .173 | -0.29 to 0.22 | |
| Medmont-Sirius | 0.04 ± 0.12 | 0.061 | -0.19 to 0.27 | |
| Topolyzer-Pentacam | -0.04 ± 0.08 | ＜.01 | -0.208 to 0.119 | |
| Topolyzer-Sirius | 0.02 ± 0.05 | ＜.01 | -0.071 to 0.121 | |
| Pentacam-Sirius | 0.07 ± 0.09 | ＜.01 | -0.1 to 0.239 | |
| SD = standard deviation, LoA = limits of agreement. | | | | |
