# Supplementary material for: A Comprehensive Assessment of the Precision and Agreement of Anterior Corneal Power Measurements Obtained Using 8 Different Devices
Source: PLoS One. 2012 Sep 25;7(9):e45607. doi: 10.1371/journal.pone.0045607 (PMC3458095; doi:10.1371/journal.pone.0045607)
Supplement: Checklist S1 — CONSORT Checklist. (DOC) [file pone.0045607.s010.doc]

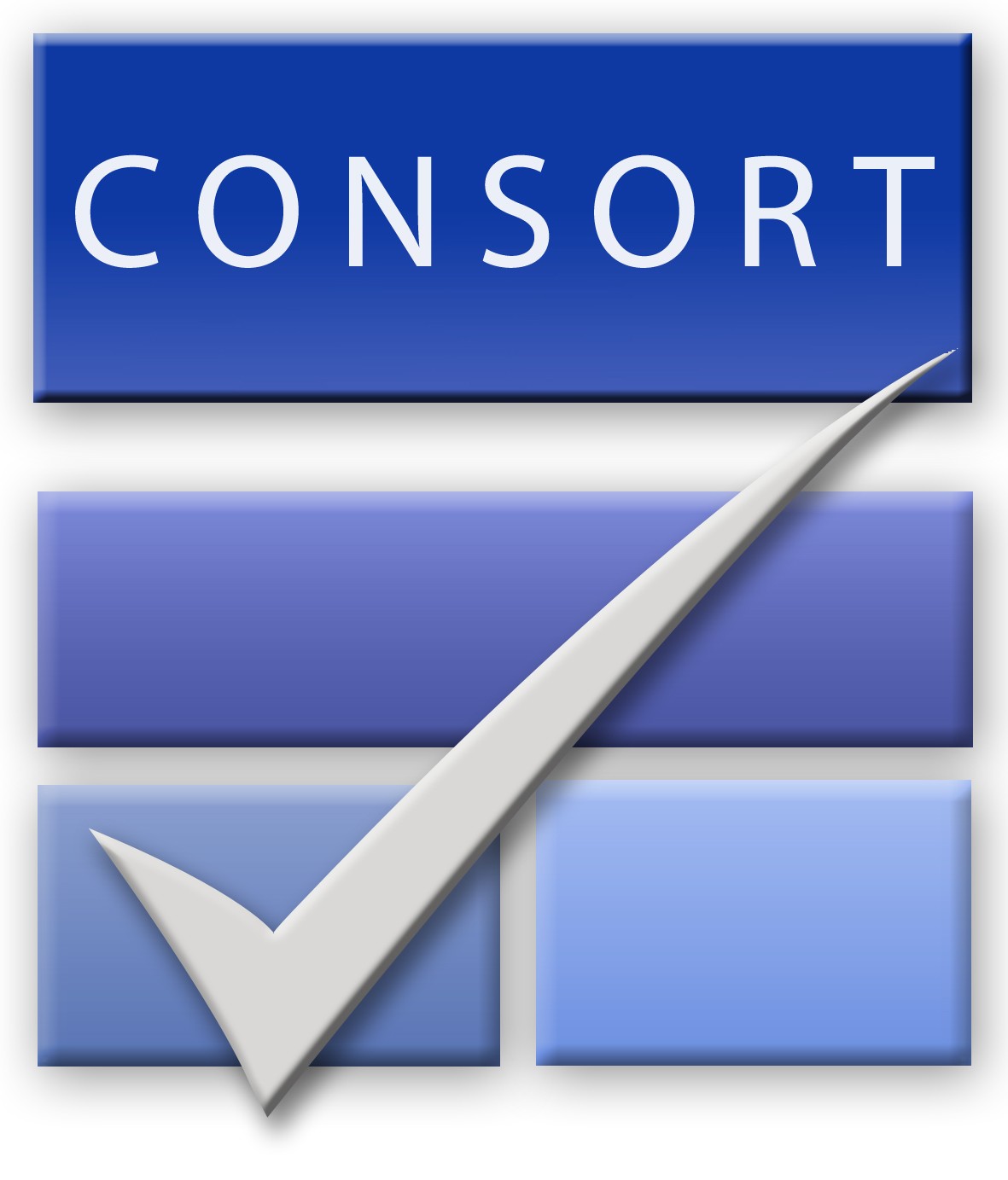
 CONSORT 2010 checklist of information to include when reporting a randomised trial

| **Paper Section and topic** | Item | **Descriptor** | **Section reported in** |
| --- | --- | --- | --- |
| Title & abstract | 1 | How participants were allocated to interventions (e.g., "random allocation", "randomized", or "randomly"). | Abstract |
| Introduction background | 2 | Scientific background and explanation of rationale. | Introduction |
| Methods participants | 3 | Eligibility criteria for participants and the [settings and locations where the data were collected](http://www.consort-statement.org/index.aspx?o=1017#3b). | Methods |
| Interventions | 4 | Precise details of the interventions intended for each group and how and when they were actually administered. | Methods |
| Objectives | 5 | Specific objectives and hypotheses. | Purpose Introduction |
| Outcomes | 6 | Clearly defined primary and secondary outcome measures and, when applicable, any methods used to enhance the quality of measurements (e.g., multiple observations, training of assessors). | Outcomes (Methods) |
| Sample size | 7 | How sample size was determined and, when applicable, [explanation of any interim analyses and stopping rules](http://www.consort-statement.org/index.aspx?o=1024#7b). | Sample Size Methods |
| Randomization -- Sequence generation | 8 | Method used to generate the random allocation sequence, including details of any restrictions (e.g., blocking, stratification) | Methods |
| Randomization -- Allocation concealment | 9 | Method used to implement the random allocation sequence, clarifying whether the sequence was concealed until interventions were assigned. | Methods |
| Randomization -- implementation | 10 | Who generated the allocation sequence, who enrolled participants, and who assigned participants to their groups. | Methods |
| Blinding (masking) | 11 | Whether or not participants, those administering the interventions, and those assessing the outcomes were blinded to group assignment. | Methods |
| Statistical methods | 12 | Statistical methods used to compare groups for primary outcome(s); [Methods for additional analyses](http://www.consort-statement.org/index.aspx?o=1029#12b), such as subgroup analyses and adjusted analyses. | Statistical analysis Methods |
| Results Participant flow | 13 | Flow of participants through each stage. Specifically, for each group report the numbers of participants randomly assigned, receiving intended treatment, completing the study protocol, and analyzed for the primary outcome. [Describe protocol deviations from study as planned, together with reasons](http://www.consort-statement.org/index.aspx?o=1086). | Methods and Results |
| Recruitment | 14 | Dates defining the periods of recruitment and follow-up. | Methods |
| Baseline data | 15 | Baseline demographic and clinical characteristics of each group. | Results and Methods |
| Numbers analyzed | 16 | Number of participants (denominator) in each group included in each analysis and whether the analysis was by "intention-to-treat". State the results in absolute numbers when feasible (e.g., 10/20, not 50%). | Results and Methods |
| Outcomes and estimation | 17 | For each primary and secondary outcome, a summary of results for each group, and the estimated effect size and its precision (e.g., 95% confidence interval). | Results |
| Ancillary analyses | 18 | Address multiplicity by reporting any other analyses performed, including subgroup analyses and adjusted analyses, indicating those pre-specified and those exploratory. | N/a |
| Adverse events | 19 | All important adverse events or side effects in each intervention group. | Results |
| Discussion interpretation | 20 | Interpretation of the results, taking into account study hypotheses, sources of potential bias or imprecision and the dangers associated with multiplicity of analyses and outcomes. | Discussion |
| Generalizability | 21 | Generalizability (external validity) of the trial findings. | Discussion |
| Overall evidence | 22 | General interpretation of the results in the context of current evidence. | Discussion |

**www.consort-statement.org**
