# Supplementary material for: A Comprehensive Assessment of the Precision and Agreement of Anterior Corneal Power Measurements Obtained Using 8 Different Devices
Source: PLoS One. 2012 Sep 25;7(9):e45607. doi: 10.1371/journal.pone.0045607 (PMC3458095; doi:10.1371/journal.pone.0045607)
Supplement: Protocol S1 — Trial Protocol. (DOC) [file pone.0045607.s011.doc]

# Study Protocol

**Study Title :** A comprehensive assessment of the precision and agreement of anterior corneal power measurements obtained using 8 different devices

**1.Review Board:**

Board Name: Wenzhou Medical College Research Review Board

Board Affiliation: Wenzhou Medical College

**2. Study Purpose**

(1)To assess the repeatability and reproducibility of anterior corneal power measurements using 8 different devices.

(2) To assess the agreement of anterior corneal power measurements using 8 different devices.

**3. Study Background**

The ability to determine corneal curvature with a high degree of accuracy and reliability is important in both clinical and research conditions. Corneal power is essential in calculating intraocular lens (IOL) power in patients undergoing cataract extraction because misleading or invalid corneal power measurement could cause inaccuracy of the IOL power and lead to a poor visual outcome. This approach provides crucial information for the screening and management of corneal refractive surgeries, especially in differentiating keratoconus and pellucid marginal degeneration. Keratometry also plays an important role in designing, monitoring and assessing the fit of orthokeratology and customized contact lenses. Although a number of studies have been performed to investigate the reliability and accuracy of corneal power measurements obtained using different technologies, such as manual or automated keratometry, computerized videokeratography, raster-stereogrammetry, slit-scanning tomography, rotating Scheimpflug tomography and optical coherence tomography, to the best of the our knowledge, no study has assessed the intrasession and intersession precision of corneal power measurements acquired by 8 different devices (three Autorefractor Keratometers (Tomey RC-5000 (Tomey Inc., Nagoya, Japan), Topcon KR-8000 (Topcon Corp., Tokyo, Japan), IOLMaster (Carl Zeiss, Jena, Germany)), three Placido disk–based corneal topographers (Medmont E300 (Medmont Pty. Ltd., Victoria, Australia), Allegro Topolyzer (WaveLight Technologie AG [Alcon Laboratories], Erlangen, Germany), EyeSys Vista (EyeSys INC., Texas, USA)), a single-rotating Scheimpflug imaging device (Pentacam, Oculus, Wetzlar, Germany), and a new single-rotating-Scheimpflug-single-Placido hybrid analyzer (Sirius, Costruzione Strumenti Oftalmici, Italy) on the same eye under the same clinical setting. No previous study has simultaneously examined the interchangeability among these devices.

**4. Study Start Date:**  April 2011

**Primary Completion Date:** June 2011

**Study Completion Date:**  July 2011

**5. Study Design**

Observational Study Model: Cohort

Time Perspective: Prospective

**6. Study Population**

Volunteers with healthy corneas

7. **Eligibility Criteria**

**(1)** **Inclusion Criteria**

- - - healthy cornea,
    - best corrected distance visual acuity (BCVA) equal to or better than 20/25,
    - willing and able to comply with scheduled visits and other study procedures.
    - Age: between 18-55years

**(2) Exclusion Criteria**

- - - History of refractive surgery or any Keratoplasty procedure,
    - use of contact lenses,
    - history of corneal dystrophies or degenerative diseases,
    - history of glaucoma or ocular hypertension,
    - significant subjective dry eye symptoms, Schirmer I test results of less than 5.0 mm, tear film break-up time shorter than 5 seconds and corneal fluorescein staining positive.

**8. Informed consent**

Patients who are eligible and not excluded according to the eligibility criteria described above will be informed of the background, purpose, method, spontaneous participation, withdraw rights of the study, and gave written consent.

**9. Privacy of study subjects**

Data obtained in the study will be handled anonymously.

**10. Study Procedures**

(1) Definition

The study’s definitions of reproducibility, repeatability and agreement were based on those adopted by the British Standards Institute and the International Organization for Standardization.

(2) Randomization

Method used to generate the random sequence by MedCalc Statistical Software. The software program is GENERATE RANDOM SAMPLE.

(3)Instruments

- 3 autorefractor keratometers
- Tomey RC-5000 (Tomey Inc., Nagoya, Japan),
- Topcon KR-8000 (Topcon Corp., Tokyo, Japan),
- IOLMaster (Carl Zeiss, Jena, Germany)
- 3 Placido disk–based corneal topographers
- Medmont E300 (Medmont Pty. Ltd., Victoria, Australia),
- Allegro Topolyzer (WaveLight Technologie AG [Alcon Laboratories], Erlangen, Germany),
- EyeSys Vista (EyeSys INC., Texas, USA),
- 1 single-rotating Scheimpflug imaging device
- Pentacam (Oculus, Wetzlar, Germany)
- 1 new single-rotating-Scheimpflug-single-Placido hybrid analyzer
- Sirius (Costruzione Strumenti Oftalmici, Florence, Italy)

(4) The measurements were collected at least 3 hours after subjects woke from sleep, between 10 am and 5 pm.

(5)The subjects were asked to avoid substantial reading prior to the measurements.

(6) Only the right eye of each subject was selected.

(7)The Cycloplegic drugs were not used.

(8) During the first session, three sets of measurements with all the devices were performed by a single experienced examiner.

(9) The examiner and subject were masked to the results of the previous measurements obtained from each device.

(10) Subjects were instructed to blink completely just before each measurement. The subjects were asked to sit back after each repeat measurement, and the device was realigned before each measurement.

(11) Measurements were repeated in the second session scheduled 1 to 2 weeks later, at almost the same time as the first session, by the same examiner using the same protocol.

**11. Outcome Measures**

(1) Flat corneal power: Kf

(2) Steep corneal power: Ks

(3) Mean corneal power: Km

(4) Vector power J0: J0 = − [cylinder/2] cos[2 × axis]

(5) Vector power J45: J45 (= − [cylinder/2] sin[2 × axis])

**12. Analysis**

(1) Repeatability and reproducibility

- within-subject standard deviation (Sw),
- test-retest repeatability (TRT),
- within-subject coefficient of variation (COV),
- intraclass correlation coefficients (ICC)

(2) Comparison among devices

- Repeated-measures analysis of variance (ANOVA) with Bonferroni correction
- Bland-Altman analysis：95% limits of agreement (LoA) as the mean difference ± 1.96 SD

**13. Handling of study data**

Data will be collected and analyzed by the principal investigator and co-principal investigator.

**14. Registration of the study**

The study will be registered in http://clinicaltrials.gov/.

**15. Publication of the study**

The study will be published to the journal under the names of the investigators, or the name of study group if necessary.
